# Polymorphic erythema associated with mycoplasma pneumoniae

**DOI:** 10.11604/pamj.2020.36.224.24439

**Published:** 2020-07-28

**Authors:** Leila Debono, Naima El Hafidi

**Affiliations:** 1 Department of Pediatrics I, Children's Hospital of Rabat, Rabat, Morocco

**Keywords:** Polymorphic erythema, mycoplasma, rash

## Image in medicine

Male child, 6 and a half years old, followed for psychomotor delay from the age of 2 years with diagnosis of epilepsy put on sodium valproate for 3 years and in the absence of seizure control carbamazepine was added 2 months before admission. The patient had presented 5 days before his consultation a generalized erythematous rash with edemas of two lower limbs, a dry cough and a fever. He was initially treated as a meningococcemia. On skin examination: generalized erythematous lesions with a rosette appearance, vesicular lesions in the lips. On admission to the service, the patient was put on acilovir, josamycin with discontinuation of carbamazepine. HSV 1 and 2 serology returned negative. Mycoplasma serology was positive for IgM and negative for IgG. The chest X-ray was normal. The skin biopsy was in favor of superficial dermatitis. The evolution was favorable with progressive disappearance of the lesions. Aciclovir was discontinued due to the rapid improvement and negativity of serology and josamycin was discontinued after 14 days of treatment. The general condition improved in 48 hours and the skin lesions disappeared in 15 days.

**Figure 1:**
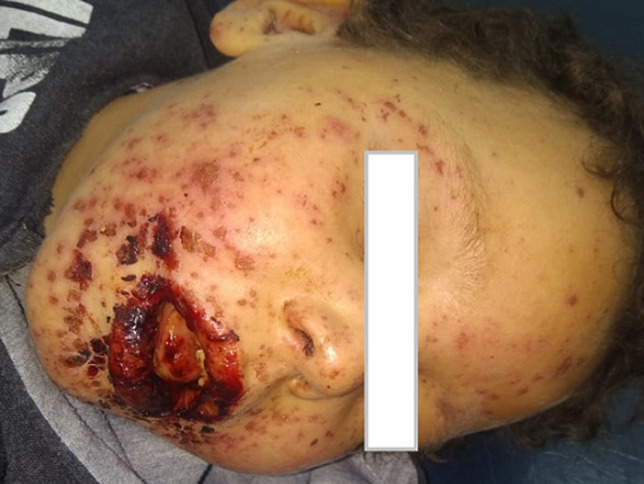
polymorphic erythema associated with mycoplasma pneumoniae in a child of six and half years old

